# Lgr5 is a marker for fetal mammary stem cells, but is not essential for stem cell activity or tumorigenesis

**DOI:** 10.1038/s41523-017-0018-6

**Published:** 2017-04-24

**Authors:** Christy L. Trejo, Gidsela Luna, Christopher Dravis, Benjamin T. Spike, Geoffrey M. Wahl

**Affiliations:** 1 Salk Institute for Biological Studies, Gene Expression Laboratory, La Jolla, CA 92037 USA; 2 Huntsman Cancer Institute, University of Utah, Salt Lake City, 84103 UT USA

## Abstract

The search for the bipotent mammary stem cells that drive mammary development requires markers to enable their prospective isolation. There is general agreement that bipotent mouse mammary stem cells are abundant in late fetal development, but their existence in the adult is vigorously debated. Among markers useful for mammary stem cell identification, the Wnt co-receptor Lgr5 has been suggested by some to be both “necessary and sufficient” for bipotency and transplantation of adult mammary stem cell activity, though other studies disagree. Importantly, the relevance of Lgr5 to the bipotency of fetal mammary stem cells has not been studied. We show here that expression of a fluorescent protein driven by the endogenous Lgr5 promoter enables significant fetal mammary stem cell enrichment. We used lineage tracing to demonstrate embryonic cells expressing Lgr5 are bipotent, while their adult counterparts are myoepithelial restricted. Importantly, our data conclusively demonstrate that Lgr5 is dispensable for both fetal and adult mammary stem cell activity and for the development of mammary tumors.

## Stem cells: protein not needed for stem cell activity in mammary gland

A protein considered necessary for mammary stem cell activity turns out to be dispensable. What’s more, it’s not required for tumor development either. Geoffrey Wahl, Christy Trejo, and colleagues from the Salk Institute for Biological Studies in La Jolla, CA, USA, investigated whether the expression of a protein called Lgr5 denotes mammary stem cells and whether it’s truly needed for the cells to be capable of giving rise to two cell lineages—a subject of active debate among researchers. Wahl’s team showed that mouse fetal mammary stem cells expressing Lgr5 had this dual-lineage capacity; their counterparts in the adult mouse did not. Yet, even though Lgr5 expression marks a population of fetal mammary stem cells, the protein is not required for stem cell activity or for tumors to form in a mouse model of breast cancer.

## Introduction

The identification of reliable markers useful for prospective identification and isolation of mammary stem cells (MaSCs) is critical for gaining a better understanding of mammary development and breast cancers that co-opt developmental and stem cell mechanisms. In this regard, markers with functional relevance would be extremely valuable, as they might also serve as therapeutic targets in specific cells or molecular processes. This has prompted many studies in a variety of tissues to obtain stem cell markers of potential functional relevance.

Wnt signaling plays a functional role in epithelial stem cell biology, and has been shown to be critical for early stages of mammary development and breast cancer.^1–4^ Wnt signaling has also been suggested to play a role in MaSCs.^5–8^ Lgr5, a G-protein-coupled receptor involved in canonical Wnt signaling, was proposed as a marker for adult MaSCs, but other reports do not support this conclusion.^9–12^

It is possible that the ongoing debate in the scientific literature concerning the role of Lgr5 in MaSC identification and function actually relates to the deeper question of whether bipotential MaSCs exist in sufficient numbers to measure accurately after birth. Studies that used lineage tracing to detect bipotent MaSCs in the adult have presented different conclusions, even when using the same Cre driver strains, including one strain in which Cre was activated from the endogenous Lgr5 locus.^7, 10–12^

In contrast to the adult mammary gland, several reports have produced a consistent view that the embryo contains bipotent cells that diminish, disappear, or become lineage-dedicated progenitors after birth.^10, 13, 14^ We therefore chose to focus on determining whether Lgr5 is a marker for fetal mammary stem cells (fMaSCs), as we previously observed that Lgr5 is expressed in mammary rudiments that harbor robust bipotent stem cell activity.^13^ We also re-examined its role as a marker for adult MaSCs in the mature adult gland. Finally, we tested the functional requirement for the Lgr5 protein and the related Lgr4 protein in mammary development and tumorigenesis through genetic ablation. Our findings demonstrate that Lgr5 serves as an enrichment marker for fMaSC activity and that Lgr5-expressing cells in the embryo can give rise to both the myoepithelial and luminal cell lineages. Further, genetically eliminating functional Lgr5 protein did not measurably affect development of the mammary rudiment, MaSC activity, or the establishment of tumors in a model of basal breast cancer. Further, elimination of functional Lgr4 had no impact on fetal mammary development or stem cell activity.

## Results

### Contrasting expression of Lgr5 in the fetal and adult mammary gland

**Fig. 1:**
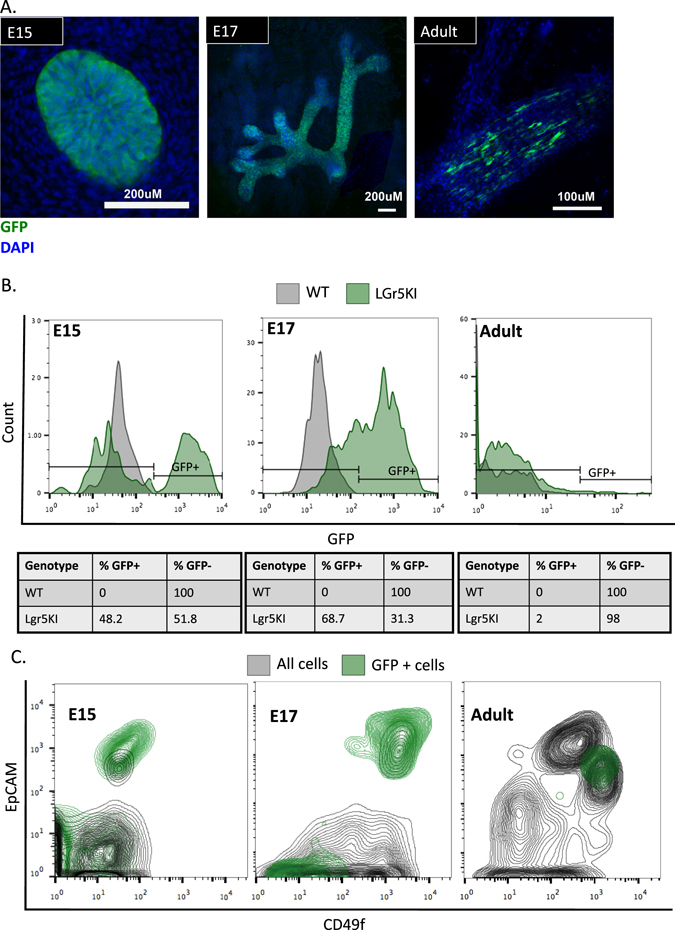
Lgr5 expression profiled in the mammary gland throughout development. **a** Mammary glands were isolated from Lgr5KI embryos at embryonic days 15 and 17 (E15 and E17) and from Lgr5KI virgin adults. Whole mounts were immunostained for GFP (*green*) and DAPI (*blue*), and analyzed on a confocal microscope. **b** Single-cell suspensions were prepared from the mammary glands of Lgr5KI mice at stages E15, E17, and adult and stained for DAPI and EpCAM. Viable (DAPI negative), epithelial (EpCAM positive) cells were then measured for GFP expression through FACS analysis. The percentage of cells that were GFP positive (*green*) was measured by designating a threshold above which the epithelial cells of the wild-type littermates (*gray*) measured zero. **c** Viable single cells were sorted for GFP expression using the parameters mentioned in Fig. 1b and analyzed for expression of EpCAM and CD49f

In order to understand the role of Lgr5 as a marker for MaSC activity, we first profiled the dynamics of its expression at time points in development at which quantitation of stem cell activity showed dramatic differences.^13^ We used a genetically engineered mouse with a modified Lgr5 allele (Lgr5KI). This mouse harbors eGFP inserted immediately downstream of the endogenous Lgr5 promoter, effectively inactivating the endogenous gene.^15^ We performed immunofluorescent staining using an antibody against GFP in whole mount mammary glands from embryonic stages 15 (E15), 17 (E17), and adult virgin mice. In both embryonic stages, Lgr5 expression is widespread, as evidenced by abundant GFP expression throughout the gland. However, in the adult virgin gland, Lgr5^POS^ cells are rare. Using the lymph node to separate nipple proximal and distal regions, we only found GFP^POS^ cells in the nipple proximal region of the gland, in agreement with previous reports^9, 10^ (Fig. 1a).

To quantify the percentage of Lgr5^POS^ cells at each of these stages, we generated single-cell suspensions from these glands and measured GFP expression in EpCAM^POS^ (epithelial) cells by flow cytometry. At the E17 and adult stage, GFP expression fell along an intensity spectrum without distinct positive and negative populations. Thus, wild-type littermates of the same stage were used to establish a threshold for delineating GFP positive and negative cells for quantification. At E15, 48.2% of epithelial cells express Lgr5, while at E17 68.7% were Lgr5^POS^. In contrast, only 2% of epithelial cells expressed Lgr5 at the adult stage (Fig. 1b). In both embryonic stages, Lgr5^POS^ cells were found in the fMaSC-containing EpCAM^HIGH^; CD49f^HIGH^ population, but a significant fraction was also present in the non-epithelial (EpCAM^NEG^) population. In the adult, all Lgr5^POS^ cells were found exclusively in the myoepithelial compartment (EpCAM^MED^; CD49f^HIGH^) (Fig. 1c). Our observations agree with previous studies showing that Lgr5-expressing cells become predominantly restricted to the myoepithelial compartment between birth and maturity.^9–11^

### Lgr5 expression marks a fetal mammary epithelial cell population enriched for stem cell activity

We next measured the stem cell activity in the Lgr5^POS^ and Lgr5^NEG^ populations revealed by our expression analyses at these different stages. We previously found that 3D culture in the presence of Matrigel provides a reliable in vitro surrogate assay for stem/progenitor activity. Under our conditions, a single MaSC clonally expands to form spheres containing basal myoepithelial cells surrounding an inner luminal layer to create a structure resembling the cell polarization seen in the native adult mammary gland.^13, 16^

**Fig. 2:**
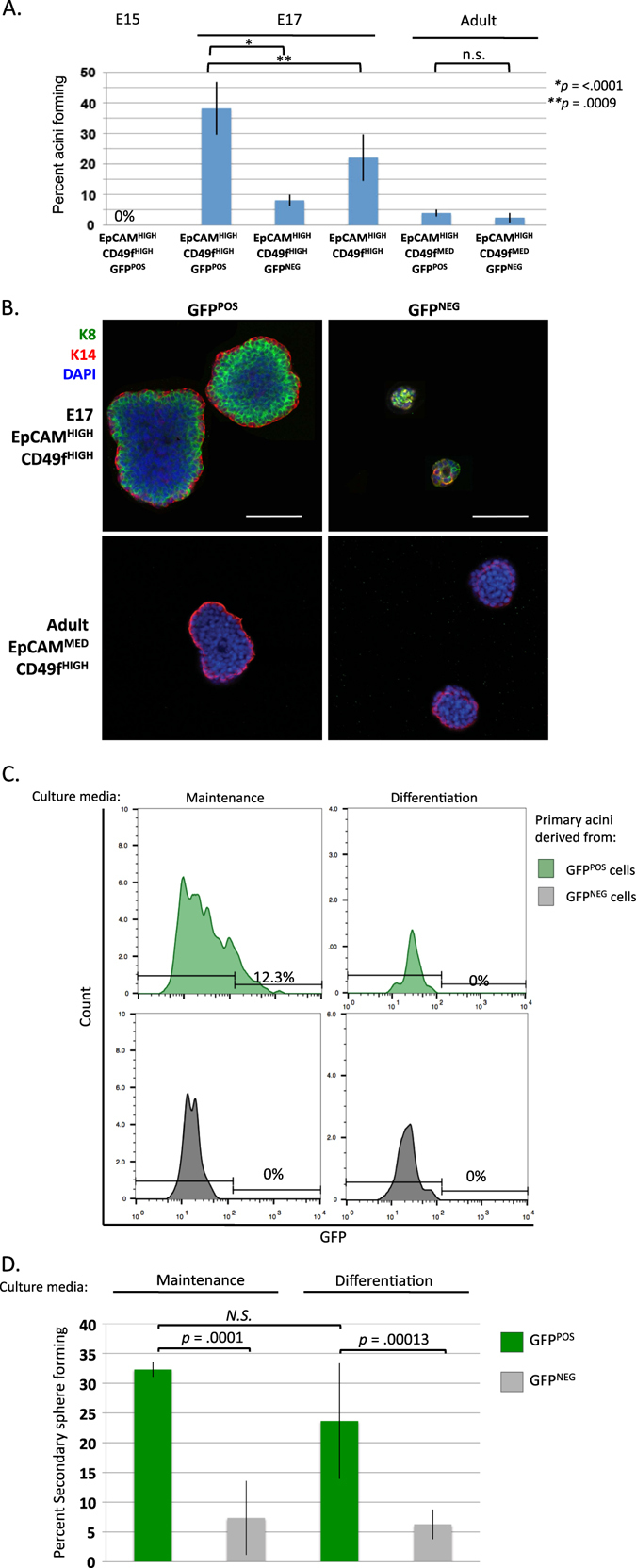
Lgr5 expression enriches for fMaSC activity in vitro. **a** Single cells were prepared from mammary glands of Lgr5KI mice at stages E15, E17, and adult, and wild-type mice at E17. EpCAM^POS^; CD49f^ POS^ cells were then sorted based on GFP expression and plated in 3D culture media containing 2% Matrigel. After 1 week, the percent of SFCs was determined by the number of spheres counted per the number of cells plated. Percent SFC is reported as averages of biological replicates and error bars represent standard deviations from five separate experiments, each with at least three technical replicates. **b** GFP^POS^ and GFP^NEG^ colonies from stages E17 and adult were stained for Keratin 8 (K8, *green*), Keratin 14 (K14, *red*), and DAPI (*blue*). Bar represents 100 μM. Images are representatives of 20 spheres for each genotype over two separate experiments. **c** Primary spheres derived from GFP^POS^ or GFP^NEG^ cells in the E17 rudiment and cultured in either maintenance or differentiation media were dissociated into single cells and live cells were measured for GFP expression using FACS. The percentage of cells that were GFP positive (*green*) was measured by designating a threshold above which cells dissociated from wild-type spheres measured zero, and is the average percentage of two separate experiments. **d** Dissociated primary spheres described in **c** were serially re-plated into their respective media and secondary sphere formation was quantified 7 days later. Percent SFC is reported as averages of biological replicates and error bars represent standard deviations from three separate experiments, each with at least three technical replicates

We sorted Lgr5^POS^ and Lgr5^NEG^ EpCAM^POS^; CD49f^POS^ mammary epithelial cells from heterozygous Lgr5KI mice to measure the frequency of sphere-forming cells (SFC) in each population at stages E15, E17, and the adult. Cells were plated at low density in 3D culture containing 2% Matrigel, and allowed to expand to generate polarized spheres. We initially used media (referred to as “maintenance media” below) containing serum and B27 supplement, as growth under these conditions produces a higher percentage of bilineage spheres than in a more restrictive, serum-free media that induces differentiation (see below).^13, 17^ In agreement with previous studies, no spheres formed from embryonic mammary epithelial cells isolated from E15, regardless of their Lgr5 status as determined by GFP expression.^13^ At E17, the SFC frequency increased significantly, with Lgr5-expressing cells (GFP^POS^) having an almost five-fold higher frequency than Lgr5 non-expressing (GFP^NEG^) cells (38.2 vs. 8%, respectively). This difference was statistically significant (*p*< .0001, Student’s *t*-test) (Fig. 2a). Colonies were co-immunostained for Keratins 8 and 14 (K8 and K14). Spheres formed from GFP^POS^ cells were distinctly polarized, with K14^POS^ cells in the myoepithelial layer and K8^POS^ cells in the luminal layer, indicating differentiation to both lineages reminiscent of adult mammary gland architecture. Spheres derived from GFP^NEG^ cells lacked polarity and the stereotypical expression of Keratins found within spheres initiated by fMaSCs (Fig. 2b). When EpCAM^HIGH^; CD49f^HIGH^ fMaSCs were sorted from wild-type littermates, the SFC frequency was about 50% of that obtained by using Lgr5 expression (i.e., GFP^POS^) as an additional marker (Fig. 2a). This indicates that the E17 Lgr5-expressing population constitutes a more selective, and more fMaSC-enriched sub population than that obtained with EpCAM and CD49f sorting alone (*p* = 0.0009). We also sorted GFP^POS^ and GFP^NEG^ cells from the EpCAM^MED^; CD49f^HIGH^ population at the adult stage, and measured SFC frequency to be 4 and 2.4%, respectively. In both cases, the majority of colonies were polarized with K14-expressing myoepithelial cells, but lacked luminal K8-expressing cells. These data demonstrate that Lgr5 expression in the adult population does not significantly enrich for sphere-forming efficiency (*p* = 0.29) (Fig. 2a, b), and are also in agreement with previously published lineage-tracing studies showing that adult Lgr5-expressing cells were predominantly restricted to a myoepithelial fate.^10, 12^

We next tested sphere formation from E17 fMaSCs in the differentiation-inducing, serum-free media (referred to as differentiation media below).^13, 16^ FACS-sorted EpCAM^HIGH^; CD49f^HIGH^ cells were further fractionated based on Lgr5 expression. Importantly, even in differentiation media, fetal-derived Lgr5 reporter-expressing cells formed spheres four-fold more efficiently than their counterparts lacking Lgr5 reporter expression (33 and 7%, respectively). These values did not differ significantly from those measured in the more permissive maintenance media (*p* = .72 for GFP^POS^, *p* = .06 for GFP^NEG^). However, spheres generated in this restrictive media were smaller in diameter than those grown in maintenance media (27.1 vs. 44.6 μM, *p* = .0002), *data not shown*. Furthermore, we noticed that spheres formed in maintenance media had fewer K8-expressing cells, which were commonly located near the basal layer, often co-staining with K14 (*white arrow*, Supplementary Fig. S1).

Primary spheres were dissociated and re-plated to test secondary sphere formation, which has been typically used as a metric of self-renewal, but may be more accurately viewed as a measure of the preservation of competence for multi-lineage differentiation. In maintenance media, 12.3% of dissociated cells from spheres that were derived from Lgr5^POS^ cells retained Lgr5 expression. This indicates that the presence of factors in maintenance media maintain Lgr5 expression. The population as a whole had a secondary SFC% of 32.3. Dissociated cells from spheres formed from GFP^NEG^ cells had a secondary SFC% of 7.3 and did not contain GFP^POS^ cells. These data suggest that Lgr5 expression in the rudiment may contribute to or correlate with the preservation of competence for multi-lineage differentiation. Importantly, the formation of secondary spheres from the Lgr5^NEG^ population was not the result of their acquisition of Lgr5 expression during primary sphere formation, as all cells in the primary spheres remained Lgr5 negative. Interestingly, cells from primary spheres derived from GFP^POS^ cells and grown in differentiation-inducing media had no detectable GFP expression upon dissociation (Fig. 2c). This suggests that factors present in maintenance media, but not in differentiation-inducing media, may activate or maintain Lgr5 expression. When primary spheres grown in differentiation-inducing media were serially passaged, secondary sphere-forming efficiency was 23.7 and 6.25% for GFP^POS^ and GFP^NEG^, respectively (Fig. 2c, d). Thus, GFP^POS^ cells isolated from the rudiment maintained the capacity to give rise to a higher fraction of secondary spheres than GFP^NEG^ cells in both maintenance and differentiation-inducing media (Fig. 2d).

We next performed mammary reconstitution assays, a method to assay stem cell potential in vivo by measuring the frequency of mammary repopulating units (MRU) in a given cell population.^18, 19^ EpCAM^HIGH^; CD49f^HIGH^ cells were sorted for GFP expression from E17/18 Lgr5KI mammary rudiments and injected in limiting dilutions into de-epithelialized fat pads of prepubescent mice. The MRU frequency in the GFP^POS^ population was 1/51 (Table 1A), which was not significantly different from that of the GFP^NEG^ population (1/53, *p* = .94) (Table 1B). This is surprising in light of a prior study indicating no MRU activity in the GFP^NEG^ fraction, but is consistent with another study.^9, 11^ The presence of only one functional Lgr5 allele in these LGR5-reporter heterozygous cells had no impact on cell engraftment and outgrowth, as we observed similar engraftment rates from the transplantation of 50 or 100 EpCAM^HIGH^; CD49f^HIGH^ cells derived from Lgr5KI and wild-type E17 littermates without sorting for GFP expression (*data not shown*).

**Table 1:** LDTA on mammary epithelial cells sorted based on Lgr5 expression

A		
E17: EpCAM^HIGH^; CD49f^HIGH^; GFP^POS^		
Cells	Fat pads injected	Fat pads reconstituted
10	5	3
12	5	1
20	5	1
25	5	2
40	3	2
50	8	5
100	7	5
250	1	1
400	3	3
500	2	2
Estimated MRU: 1/51.5		

B		
E17: EpCAM^HIGH^; CD49f^HIGH^; GFP^NEG^		
10	5	1
12	2	0
25	2	0
30	3	0
40	3	2
50	1	1
100	5	5
300	3	3
Estimated MRU: 1/53.2		

C		
Adult: EpCAM^MED^; CD49f^HIGH^; GFP^POS^		
20	4	0
50	6	1
100	6	6
250	2	2
500	3	2
Estimated MRU: 1/141		

D		
Adult: EpCAM^MED^; CD49f^HIGH^; GFP^NEG^		
50	1	0
200	5	1
400	9	2
800	4	2
Estimated MRU: 1/1289		

We also measured the MRU frequency of adult mammary cells based on Lgr5 expression. We transplanted 20–500 EpCAM^MED^; CD49f^HIGH^; GFP^POS^ adult cells from virgin Lgr5KI mice, and found the MRU frequency was ~1 MRU in 141 cells, a significant decrease compared with the analogous population isolated from the embryonic stage (Table 1C) (*p* = .01). We also transplanted EpCAM^MED^; CD49f^HIGH^; GFP^NEG^ adult cells in limiting dilution and estimated the MRU fraction to be 1/1289 (Table 1D). Thus, Lgr5 expression enriches for adult MRU but does so much less effectively than previously reported. However, as there are far more Lgr5^NEG^ than Lgr5^POS^ cells in the adult gland (98 vs. 2%), we estimate that there are approximately five times as many Lgr5^NEG^ MRU’s as Lgr5^POS^ MRU’s.

### Lgr5-expressing cells in the embryo have a bipotent fate

**Fig. 3:**
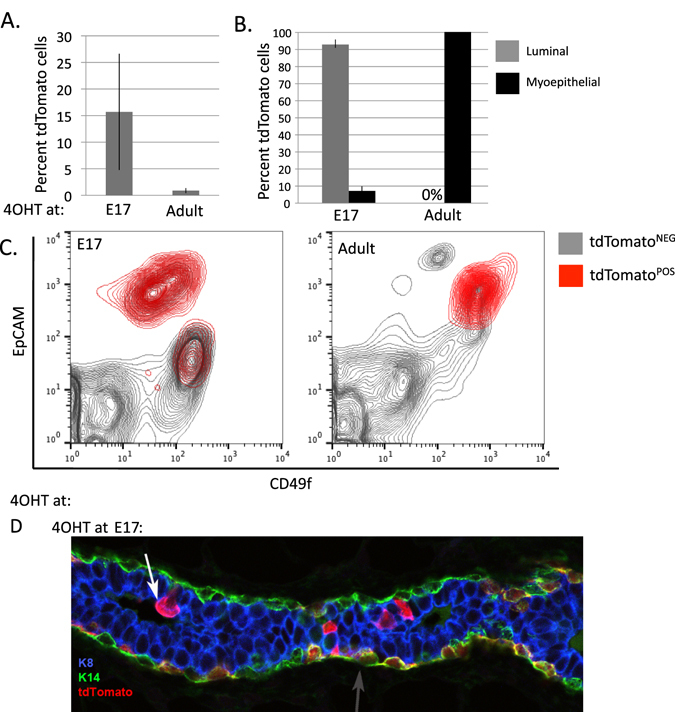
Lgr5-expressing cells are bipotent in the embryo and lineage restricted in the adult. **a**–**c** Lgr5KI; R26^LSL-Tom^ were injected with 4OHT at stage E17 and adult. Eight weeks later, single-cell suspensions were prepared and viable (DAPI negative) cells were analyzed for expression of the tdTomato fluorescent reporter, EpCAM, and CD49f. The percentage of cells that were tdTomato positive was measured by designating a threshold above which epithelial cells of R26^WT^ measured zero. **d** Lgr5KI; R26^LSL-Tom^ pregnant mice were injected with 4OHT at stage E17. Eight weeks later, they were harvested, fixed, sectioned, and stained for Keratin 8 (K8, *blue*), Keratin 14 (K14, *green*), and tdTomato (*red*). tdTomato co-staining with K8-expressing luminal cells and K14-coexpressing myoepithelial cells are indicated by the *white and gray arrows*, respectively

We next performed lineage tracing to determine the fate of Lgr5-expressing cells in the embryonic and adult mammary glands. The Lgr5KI allele contains a 4-Hydroxytamoxifen (4OHT)-inducible Cre used to permanently turn on a fluorescent reporter through recombination-mediated removal of a stop element. We crossed the Lgr5KI model to a reporter with Cre-inducible expression of the tdTomato fluorescent protein from the Rosa26 locus (R26^LSL-Tom^).^20^ Lgr5KI; R26^LSL-Tom^ mice were exposed to 4OHT, permitting the expression of tdTomato in Lgr5-expressing cells, and analyzed 8 weeks later. Lgr5-expressing cells labeled at late gestation resulted in 16% tdTomato^POS^ epithelial cells, which were found both in the myoepithelial and luminal lineages (7 vs. 93%, respectively) (Fig. 3a–c). We verified dual lineage fate by staining sectioned glands with an antibody against tdTomato and observed labeling in both K8-expressing luminal cells and K14-expressing myoepithelial cells (Fig. 3d). Labeling at the adult stage resulted in .8% tdTomato^POS^ epithelial cells, all of which were myoepithelial (Fig. 3c).

### Lgr5 function is not required for MaSC activity

**Fig. 4:**
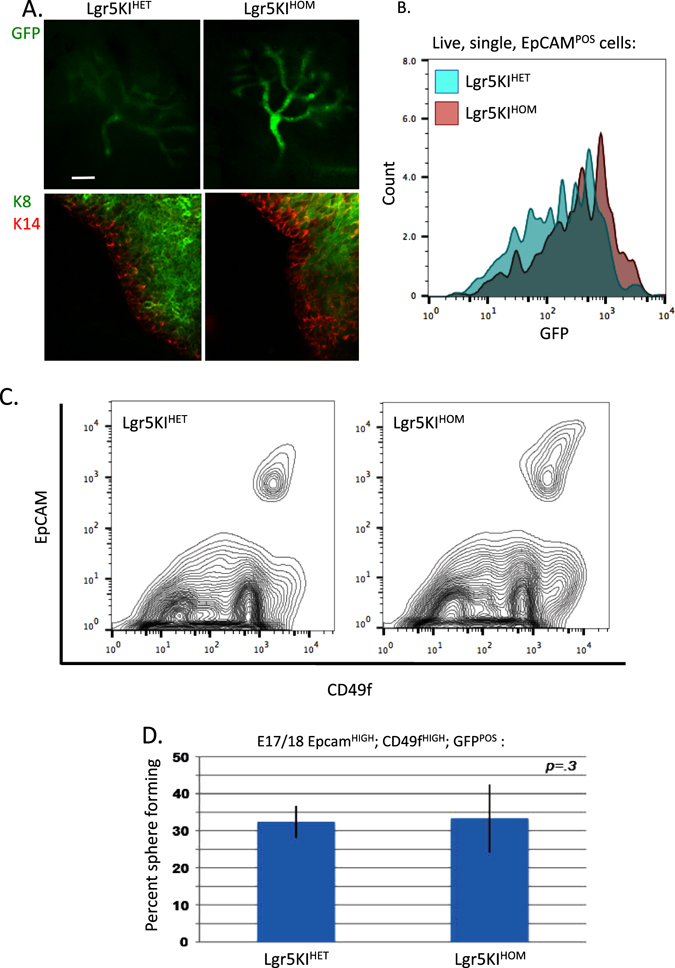
Analysis of fetal mammary rudiments heterozygous (Lgr5KI^HET^) and homozygous (Lgr5KI^HOM^) for the Lgr5KI allele. **a** Fetal mammary rudiments were isolated from Lgr5KI^HET^ and Lgr5KI^HOM^ littermates at stage E18 and stained in whole mount for GFP (*top*, bar represents 200 μM), and Keratin 8 (K8, *green*) and Keratin 14 (K14, *red*, *bottom*). **b** Single-cell suspensions were prepared from Lgr5KI^HET^ and Lgr5KI^HOM^ littermates at stage E18. Live, single, epithelial (EpCAM^POS^) cells were analyzed for GFP expression. **c** Single-cell suspensions were prepared from Lgr5KI^HET^ and Lgr5KI^HOM^ littermates at stage E18. Live, single, epithelial cells were analyzed for EpCAM and CD49f expression by FACS. **d** Single-cell suspensions were prepared from Lgr5KI^HET^ and Lgr5KI^HOM^ littermates at stage E18. Live, single, EpCAM^HIGH^; CD49f^HIGH^; GFP^POS^ cells were sorted and plated in to 3D culture containing 2% Matrigel. After 1 week, spheres were quantified and percent SFCs was determined by the number of spheres counted per the number of cells plated. Percent SFC is reported as averages of biological replicates and error bars represent standard deviations from three separate experiments, each with at least three technical replicates

The studies reported above do not address whether Lgr5 protein contributes functionally to embryonic or adult MaSC activity. Addressing this issue is critical, as Lgr5 is currently under investigation as a clinical target for treatment of cancers and other Wnt-related diseases. We therefore tested whether functional Lgr5 protein is required for normal mammary gland development and stem cell activity. We took advantage of the gene inactivation resulting from the Lgr5KI allele. Importantly, while mice homozygous for the Lgr5KI allele are born at normal Mendelian ratios, they die within 24 h of birth due to craniofacial abnormalities and other developmental defects.^21^ Homozygous embryos are viable, and easily identified from heterozygous littermates by ankyloglossia and a brighter GFP signal in the mammary buds and facial structures. Allele-specific PCR verified the genotyping of homozygous vs. heterozygous (Lgr5KI^HOM^ and Lgr5KI^HET^, respectively) embryos (Supplementary Fig. S2A). The mammary rudiments of Lgr5KI^HOM^ E17/18 embryos were indistinguishable from their heterozygous and wild-type littermates in terms of size, Keratin staining, and abundance of EpCAM^HIGH^; CD49f^HIGH^ cells. Indeed, the only discernable difference between Lgr5KI^HOM^ and Lgr5KI^HET^ mammary rudiments was an increase in the GFP signal resulting from two copies of the knock-in allele (Fig. 4a–c).

We tested whether functional Lgr5 protein was necessary for fMaSC activity in vitro. EpCAM^HIGH^; CD49f^HIGH^; GFP^POS^ cells from Lgr5KI^HET^ and Lgr5KI^HOM^ E17/18 embryos had no significant difference in the frequency of SFCs when measured in 3D culture in maintenance media (32.4 and 33%, respectively, *p* = .3) (Fig. 4d). We also compared self-renewal in Lgr5KI^HOM^ and Lgr5KI^HET^ fMaSCs and saw no difference in secondary sphere-forming capacity (28 and 30%, respectively, *p* = .3, *data not shown*). These experiments were repeated in differentiation-inducing media and revealed no difference in SFC% between Lgr5KI^HOM^ and Lgr5KI^HET^ fMaSCs, *data not shown*.

**Fig. 5:**
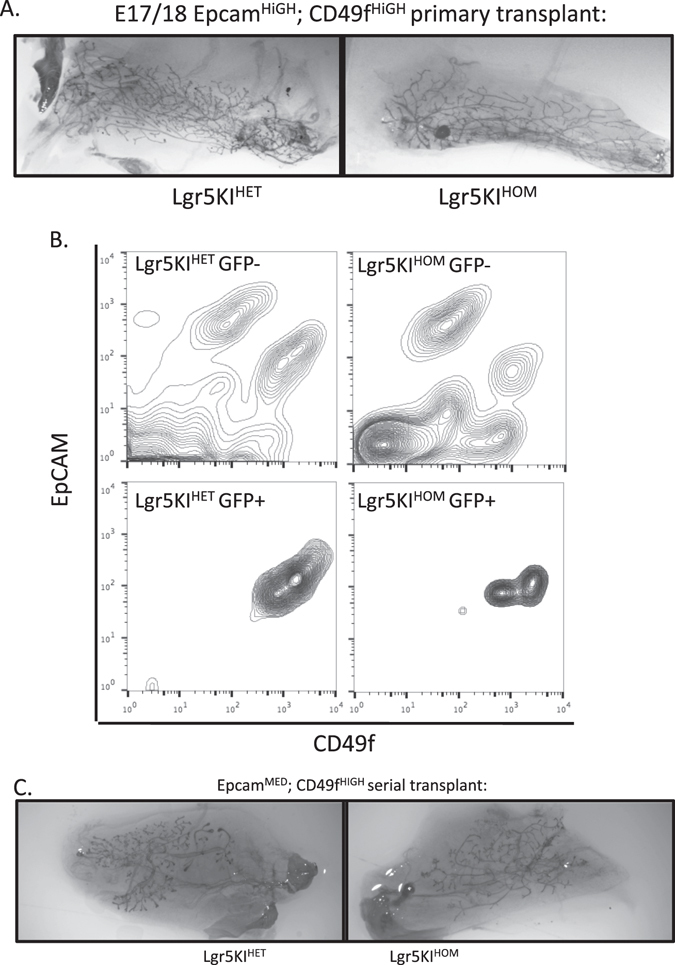
Lgr5 function is not required for mammary transplantation capacity. **a** Single-cell suspensions were prepared from Lgr5KI^HET^ and Lgr5KI^HOM^ littermates at stage E18. Live, single, EpCAM^HIGH^; CD49f^HIGH^; GFP^POS^ cells were sorted and injected into cleared fat pads of recipient mice. Eight weeks later, glands were isolated and carmine stained to visualize outgrowth and take rates were calculated as the fraction of positive outgrowths per number of injections. **b** Three thousand fMaSCs from Lgr5KI^HET^ and Lgr5KI^HOM^ littermates were transplanted into four fat pads each and outgrowths were produced as described in **a**. Outgrowths were dissociated and single cells were pooled and measured for GFP, EpCAM, and CD49f expression using FACS. **c** Four thousand cells from the EpCAM^MED^; CD49f^HIGH^ population described in Fig. 4b were re-transplanted into two recipient, cleared fat pads for each genotype. Eight weeks later, secondary transplants were carmine stained to visualize epithelium of secondary outgrowths. Images are representatives of three positive outgrowths for each genotype

We next measured the capacity of Lgr5KI^HOM^ cells to generate outgrowths upon transplantation. EpCAM^HIGH^; CD49f^HIGH^ fMaSCs from Lgr5KI^HOM^ E17/18 embryos were transplanted into cleared fat pads and isolated 8 weeks later; 2/5 outgrowths resulted from transplantation of 50 cells, and 4/4 outgrowths resulted from transplantation of 400 cells (Fig. 5a). These findings indicate that a complete absence of Lgr5 expression does not compromise fMaSC activity assayed in vivo.

We similarly asked whether the function of Lgr5 is required for adult MaSC activity. EpCAM^HIGH^; CD49f^HIGH^ fMaSCs from Lgr5KI^HOM^ and Lgr5KI^HET^ littermates were transplanted into cleared fat pads to allow for outgrowth. Eight weeks later, glands were dissociated into single cells to determine whether the transplanted cells could contribute to mammary cell lineages as analyzed by fluorescence activated cell sorting (FACS). We saw no difference in the percentage of GFP^POS^ cells or distribution of cells with regard to EpCAM and CD49f expression. Of note, cells with Lgr5 promoter activation (GFP^POS^) from either Lgr5^HOM^ or Lgr5^HET^ genotypes were restricted to the myoepithelial population (Fig. 5b). The EpCAM^MED^; CD49f^HIGH^ myoepithelial population from both Lgr5KI^HOM^ and Lgr5KI^HET^ reconstituted adult glands were then serially transplanted into cleared fat pads of secondary recipient mice. Eight weeks later, the second set of glands was dissected and positive outgrowths were noted, demonstrating that both Lgr5KI^HOM^ and Lgr5KI^HET^ cells were capable of serial transplant (Fig. 5c). We conclude that Lgr5 function is not required in vivo for fetal or adult MaSC activity as measured by transplantation.

### Lgr5 is not required for tumorigenesis

**Fig. 6:**
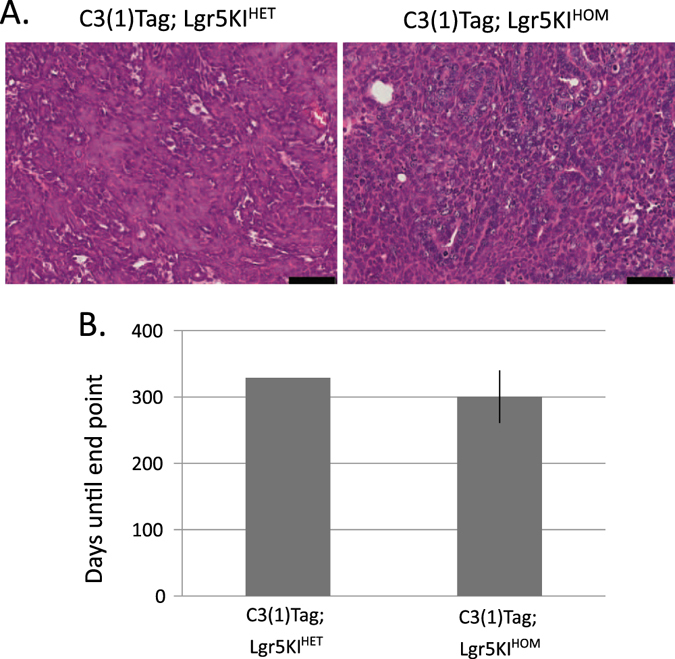
Lgr5 function is not required for mammary tumorigenesis. Four thousand EpCAM^HIGH^; CD49f^HIGH^; GFP^POS^ cells were harvested from Lgr5KI^HET^ and Lgr5KI^HOM^; C3(1)Tag mice at E18 and injected into cleared recipient fat pads. Mice were euthanized at end point, which was when tumors reached 1 cm in diameter. **a** H and E stained sections of tumors harvested from mice transplanted with C3(1)Tag; Lgr5KI^HET^ and C3(1)Tag; Lgr5KI^HOM^ fMaSCs. Bar represents 50 µM. **b** Time to endpoint (tumors with a diameter of 1 cm) was compared between glands that were transplanted with Lgr5KI^HOM^ or Lgr5KI^HET^; C3(1)Tag fMaSCs. Two mice reached end point due to Lgr5KI^HET^ C3(1)Tag fMaSCs, both at 329 days. Three mice reached end point due to Lgr5KI^HOM^ C3(1)Tag fMaSCs, at an average of 300 days (+/− 45 days)

We were also interested in whether Lgr5 contributes to tumorigenesis, as it has been suggested to be a potential therapeutic target for various tumor types and may have implications for treating basal-like breast cancers.^22–24^ We therefore ablated Lgr5 function in a mouse model of human basal-like breast cancer using the Lgr5KI strategy described above.^25, 26^ EpCAM^HIGH^; CD49f^HIGH^; GFP^POS^ cells were isolated from E18 Lgr5KI^HET^ and Lgr5KI^HOM^ embryos carrying the C3(1)TAg allele and injected into cleared fat pads of six recipient mice. Three mice were euthanized due to Lgr5KI^HOM^ fMaSC-derived tumors reaching 1 cm in diameter (end point), while two mice reached end point due to Lgr5KI^HET^-derived tumors. Tumor onset, grade, and rate of growth were not measurably different after orthotopic injection of 4000 Lgr5KI^HET^ or Lgr5KI^HOM^ cells (onset of 329 and 300 days, respectively; Fig. 6a, b). We next performed immunostaining against GFP in these tumors and found no evidence of Lgr5 promoter activation (Supplementary Fig. S3). Our results clearly demonstrate that functional Lgr5 is not required for tumorigenesis induced by the C3(1)Tag allele. If the data from this mouse model can be extrapolated to human basal-like breast cancers, we question whether inhibiting Lgr5 function will be able to significantly impact the clinical course of these cancers.

## Discussion

The mammary gland is a highly dynamic organ in which the re-establishment of tissue homeostasis following cycles of growth and involution has been assumed to be fueled by bipotential adult stem cells. However, recent studies have provided conflicting evidence for the existence of such cells, and it is now considered likely that highly proliferative, unipotent progenitors may instead serve this purpose.^10, 27^ By contrast, in vitro sphere-forming assays, limiting dilution transplantation assays, and in vivo lineage-tracing assays at embryonic time points have led to the conclusion that the mid-to-late stage fetal mammary rudiment contains an abundant source of bipotent MaSCs.^10, 13, 17, 28^ Shortly after birth, there is a significant reduction in MaSC activity, suggesting that postnatal development involves rapid lineage restriction. Importantly, fMaSCs share a striking overlap in gene expression with human triple negative basal-like breast cancers.^13^ Identifying markers to further purify fMaSCs is an important objective to enable a better understanding of the mechanisms by which the bipotent MaSC state becomes specified. Furthermore, analyses of pathways critical for fMaSC proliferation, survival, and developmental plasticity may facilitate the identification of markers for detecting occult basal-like breast cancer cells and targeted therapies to better treat basal-like breast cancers.

Lgr5 has been reported to specifically mark transplantable adult MaSCs.^9^ However, other groups found that Lgr5 specifically marks a population of nipple proximal, myoepithelial cells in the adult mammary gland and failed to find significant enrichment for mammary repopulating capacity using Lgr5 as a marker.^8, 10, 11^ Conflicting data have also been obtained using lineage tracing, where one study provided evidence that adult Lgr5-expressing cells are bipotent, while another demonstrated myoepithelial restriction.^7, 10, 12^ These disparate conclusions may arise from technical details such as timing and dose of tamoxifen, imaging strategies, statistical methodology, and differences between in vivo lineage-tracing and transplantation assays.

Given the current lack of consensus concerning the existence of bipotent adult MaSCs, we have focused on the importance of Lgr5 in the fetal mammary epithelial compartment as the existence of bipotent MaSCs within it has been unequivocally established in vitro, in vivo, and by transplantation.^10, 13, 28^ We previously found that Lgr5 is more highly expressed in mammary epithelial cells at E18 relative to E15, correlating with a dramatic increase in stem cell activity.^13^ In the present study, we show that the fMaSCs expressing elevated Lgr5 at the E17/18 stage have a significantly higher capacity to form properly polarized, multi-lineage spheres, suggesting a potential association between Lgr5 expression and stem cell activity. Nevertheless, despite expressing Lgr5, EpCAM^HIGH^; CD49f^HIGH^ cells isolated from stage E15 do not transplant efficiently as single-cell suspensions, and did not form spheres, indicating that Lgr5 is not an independent marker of stem cells and that its expression is not sufficient for stem cell activity.^13^

In contrast to the in vitro sphere-forming assay, Lgr5 expression in EpCAM^HIGH^; CD49f^HIGH^ cells isolated from E17/E18 embryos did not confer a significant stem cell enrichment in transplantation assays, as cells not expressing Lgr5 had a comparable fraction of MRUs to those that do express Lgr5. Our data are consistent with prior reports describing significant discrepancies between in vitro surrogate assays for stem cell function, and in vivo assays of the same cells. Furthermore, there are also differences between results obtained using orthotopic transplantation and lineage tracing. Potential reasons for such differences have been discussed at length.^10, 14^ As one example, cues specific to the transplantation setting may permit or promote bilineage differentiation of myoepithelial cells independent of their Lgr5 status. This interpretation, that transplantation reveals a degree of cellular plasticity that is not generally observed under normal physiological conditions, is also supported by past studies showing that cell fate can be altered through transplantation.^10, 29^ Indeed, when we transplanted E17/18 EpCAM^HIGH^; CD49f^HIGH^ cells that did not express Lgr5 (GFP^NEG^) and subsequently dissociated reconstituted glands 8 weeks later, we found GFP^POS^-expressing cells exclusively in the EpCAM^MED^; CD49f^HIGH^ population at a frequency consistent with adult endogenous glands (Supplementary Fig. S4). This indicates that Lgr5 expression is induced between transplantation and outgrowth. In contrast, Lgr5 expression was not induced in our in vitro sphere-forming assays using Lgr5^NEG^ cells, even in stem cell permissive media that promotes some degree of lineage plasticity. However, due to the small sphere size, low sphere-forming efficiency of Lgr5^NEG^ cells, and sufficient cells to perform only two passages, we cannot rule out the possibility that more extensive passaging may result in acquisition of Lgr5 expression. Further, in vitro, adult EpCAM^MED^;CD49f^HIGH^ cells predominantly formed spheres containing Keratin 14-expressing cells and lacked cells expressing Keratin 8, regardless of Lgr5 expression. These observations support the notion that EpCAM^MED^; CD49f^HIGH^ cells derived from the adult gland are more likely to be myoepithelial restricted and in vitro SFC assays may serve as a more stringent method for testing bipotent stem cell activity.

In the adult, when we measured the frequency of MRU within the EpCAM^MED^; CD49f^HIGH^ Lgr5-expressing population, our findings agreed with those of deVisser et al. and Wang et al. but were in stark contrast to the work of Plaks et al. which claimed that one in four Lgr5-expressing adult mammary epithelial cells represents an MRU.^8, 9, 11^ The latter estimate was determined in part by measuring the percentage of positive outgrowths from injecting 10, 50, and 100 cells, and then averaging this value to arrive at 27%, or 1/4 of cells representing an MRU. By contrast, extreme limiting dilution analysis (ELDA) is a statistical method appropriate for estimating stem cell frequency in a population of cells.^30^ We applied ELDA to our data set and arrived at an MRU frequency of 1/141 in the adult EpCAM^MED^; CD49f^HIGH^; Lgr5-expressing population. It is worth noting that we arrived at the exact same estimate of MRU when applying ELDA to the Plaks data set.

It has been established in our study and others that Lgr5-expressing myoepithelial cells are restricted to the nipple proximal region in the adult gland.^9, 10^ Cells originating from the embryo with long-term label retaining capacity were previously reported to be nipple proximal.^28^ We show that these cells are approximately nine-fold enriched for MRU when compared with their Lgr5 negative counterparts. It may be that this location marks cells with elevated plasticity as a function of their relatedness to fMaSCs, sustained Wnt signaling, or both. Nevertheless, the GFP^NEG^ population, which make up the majority of the EpCAM^MED^; CD49f^HIGH^ population, has a significant MRU content despite its higher degree of heterogeneity and increased proportion of cells that are unable to achieve outgrowth capacity upon transplantation. All told, our data suggest that while Lgr5-expressing myoepithelial cells are enriched for stem cell activity relative to Lgr5 negative cells, there are five-fold more Lgr5 negative transplantable stem cells per gland than Lgr5 positive ones. Thus, when one considers total cell numbers, Lgr5 expression in the adult does not enrich for stem cell activity.

Our in vitro and in vivo data support the evidence that bipotent stem cells are abundant in the embryo but are rare or non-existent in the adult. While we did find a low frequency of MRU in adult cells, we note that transplantation assays necessarily disrupt cell–cell and cell–microenvironment interactions, which may affect cell fate or induce plasticity.^10^ By contrast, lineage tracing from Lgr5-expressing embryonic cells has not been done previously, and we used this method as it preserves cellular contacts and context, which are disrupted using sphere forming or MRU transplantation analyses. Lgr5-expressing cells labeled at late gestation generated both myoepithelial and luminal lineages, while labeling in the adult traced only myoepithelial cells. Our data support previous lineage-tracing studies that show predominant myoepithelial lineage restriction in adult Lgr5-expressing cells. It is possible that the dose of 4OHT used in the adult was not sufficient to label less abundant Lgr5-expressing cells with luminal fate, but we used a significantly smaller dose for embryonic labeling, which consistently resulted in bi-lineage labeling. Previous studies that employed saturation-level doses of 4OHT in Lgr5KI; R26^LSL-Tom^ adult mice and analyzed reporter expression 48 h later detected greater than 30% of labeled cells to be luminal.^12^ We did not observe labeled luminal cells 8 weeks following a significantly lower dose of 4OHT. This may be attributed to insufficient labeling, or the particular stage of the estrous cycle the mice were in at the time of labeling. While we did not use a dose low enough to enable us to conclude that singly labeled clones at E18 gave rise to either lineage, we did find that the majority of cells traced from the embryo are luminal. This contrasts with our results from the adult. A previous study in which Lgr5-expressing cells were traced from postnatal day 1 found complete restriction to the luminal fate.^11^ Taken with our dual lineage labeling in late gestation, these data support a model in which Lgr5-expressing cells are multipotent in the embryo, become luminal fate restricted at birth, and switch to a myoepithelial fate in the adult. We cannot rule out, however, the presence of rare multipotential Lgr5-expressing cells in the adult gland with the current analysis.

Our data clearly show that complete absence of Lgr5 function does not adversely affect mammary rudiment development, and that Lgr5 loss does not affect stem cell activity measured in vitro or in vivo. This was surprising as using the Lgr5 reporter does enable significant enrichment for an fMaSC population with increased sphere-forming ability. However, the reporter merely serves as an indicator of Lgr5 promoter activation, and cannot be used as a criterion for the requirement for functional Lgr5 protein. Lgr5 promoter activation is not the same as Lgr5 protein function, and testing the requirement for the latter would involve selectively ablating Lgr5-expressing cells. Plaks et al. used a mouse that expresses diphtheria toxin receptor (DTr) driven by the Lgr5 promoter to test this possibility. Their results indicate that eliminating Lgr5-expressing cells by diphtheria toxin (DTx) administration dramatically reduced sphere formation and MRU activity, suggesting that Lgr5-expressing cells are required for mammogenesis. In order to assess whether Lgr5-expressing cells were similarly necessary for stem cell activity in the embryo, we initially carried out dose curves to test whether DTx adversely affects sphere formation in wild-type fMaSCs, a control not performed previously. In fact, we found a marked decrease in sphere formation in wild-type control fMaSCs using as little as 0.2 μg/mL DTx. Using RT-PCR, we subsequently confirmed DTr expression in wild-type fMaSCs (Supplementary Fig. S5A, B), precluding the use of DTx as a means of specifically eliminating stem cells with engineered Lgr5 promoter-driven DTr over-expression in this system.

Our data further demonstrate that functional Lgr5 is not necessary for tumorigenesis in an SV40 T-antigen-driven mammary cancer model. This result is important given the reported relationship between Lgr5 expression and stem cell activity, and because Lgr5 therapeutics are in development for various tumors where Lgr5 has been implicated as a stem cell marker. Our data clearly show that in the C3-1-Tag model, Lgr5 function is clearly unnecessary for tumor initiation or progression, and that in such a model an anti-Lgr5 therapy would not be efficacious. However, it is possible that other tumor models may require Lgr5 function, and this would need to be evaluated on a case-by-case basis. We also point out that in some cases, activation of the Wnt pathway correlates with a more differentiated phenotype, while in others Wnt activation may contribute to proliferation, the role of Wnt activity, and how Lgr5 contributes to that requires further investigation.^31^ An application of anti-Wnt therapies would require knowledge of the phenotypic contributions of Wnt function in individual tumors.

Lgr4 is another Lgr family member with functional implications for MaSC biology.^6, 32^ We conducted gene expression assays to show that Lgr4 is expressed in fMaSCs, but its expression does not increase when Lgr5 is lost (Supplementary Fig. S6A). We found that Lgr4 is not necessary for normal mammary rudiment development or stem cell activity (Supplemental Material, Supplementary Fig. S6B–D). This does not rule out that Lgr4 compensates for loss of Lgr5, as additional experiments involving dual knock out are required. Studies employing combinatorial knock-out of Lgr4 and Lgr5 found a significantly larger impact on embryonic skin development than either single knock out alone.^33^ Further, an additional family member, Lgr6, is reported to play a functional role in adult mammary progenitor activity and tumorigenesis, and may be the required for mammary rudiment development alone or in combination with the other Lgr family members.^34, 35^

Further enrichment of the fMaSC-containing population will allow for more efficient detection of pathways involved in specification of the fetal MaSC state, and holds the potential to provide novel insight into the development of therapies for basal-like breast cancer. Our studies identify Lgr5 as a valuable enrichment marker for fetal MaSCs, but they also unequivocally show for the first time that Lgr5 function is neither required for mammary gland development nor for the production or maintenance of transplantable MaSCs. Selectively sorting for Lgr5 expression in EpCAM^MED^; CD49f^HIGH^ fMaSCs leads to a two-fold enrichment in single cells able to give rise to colonies containing both luminal and myoepithelial cells. Our in vitro studies also reveal that Lgr5 does not enrich for a similarly robust or bipotent adult MaSC. This may be explained by the observation that in the adult gland, such cells are either very rare, or the adult gland is maintained by lineage restricted luminal, alveolar, and myoepithelial progenitors.

## Materials and methods

### Mice

All protocols involving animal use were reviewed and approved by an institutional animal care and use committee. Mice were housed in animal facilities with full accreditation by the Association for Assessment and Accreditation of Laboratory Animal Care. Analysis of the adult stage was done on virgin mice between postnatal day 45 and 60. Lgr5KI mice (B6.129P2-*Lgr5* ^*tm1(cre/ERT2)Cle*^/J) were purchased from Jackson Laboratory and back-crossed and maintained on the CD1 background using mice purchased from Charles River. Presence of the Lgr5KI allele and validation of homozygous genotyping was conducted with primers targeting exon 1 of the wild-type Lgr5 gene and the Lgr5KI allele. Primer sequences were provided by The Jackson Laboratory (common: 5′-CTG CTC TCT GCT CCC AGT CT-3′; wild-type reverse: 5′-ATA CCC CAT CCC TTT TGA GC-3′; mutant reverse: 5′-GAA CTT CAG GGT CAG CTT GC-3′). CB-SCID mice purchased from Charles River were used as recipient mice in transplantation studies. For tumorigenesis studies, C3(1)Tag mice (FVB-Tg(C3-1-TAg)cJeg/JegJ) were purchased from Jackson Laboratories and crossed with Lgr5KI mice. For lineage-tracing studies, R26^LSL-Tom^ mice (B6;129S6-*Gt(ROSA)26Sor* ^*tm9(CAG-tdTomato)Hze*^/J) were purchased from Jackson Laboratories and crossed with Lgr5KI mice. Lgr4KI mice were a gift from Jan Tchorz of The Novartis Institute for Biomedical Research. For experiments involving mice, at least five female embryos (50 rudiments) or four adult mice (8 glands) were processed, with experiments being repeated at least three times.

### Mammary cell isolation

Fetal mammary buds/rudiments were isolated from female embryos, pooled and digested in Epicult-B Basal Medium (Stem Cell Technologies) containing Collagenase and Hyaluronidase, and supplemented with 5% fetal bovine serum (FBS), penicillin/streptomycin, and hydrocortisone with gentle agitation at 37 °C for 90 min. From virgin 35-day old females, the number 4 mammary glands were removed, minced, and digested in the same digestion conditions for 3 h. For all stages, red blood cells were lysed with ammonium chloride and single-cell suspensions were prepared by incubating in Dispase and DNase. Adult glands were additionally incubated in .25% Trypsin-EDTA. Cell suspensions were then passed through a .40 μM filter and resuspended in Hank’s balanced salt solution containing 2% FBS and DNase. For all functional analysis performed on cells based on GFP expression, cells were gated on GFP expression using thresholds determined from wild-type littermates. For sorting, gates were shifted to the right (GFP^POS^) or left (GFP^NEG^) to eliminate cells that may have intermediate Lgr5 expression.

### Immunostaining

For FACS analysis, cells were subjected to lineage depletion using biotinylated antibodies against Ter119 (BD cat. no. 553672, 1:25), CD45 (BD cat. no. 553078, 1:25), and CD31 (BD cat. no. 553371, 1:25) followed by Streptavidin-APC-Cy7 (BD cat. no. 554063, 1:100). Cells were stained for FACS isolation with antibodies against CD49f (BioLegend cat. no. 313612, 1:250) and EpCAM (BioLegend cat. no. 118212, 1:250) and 4,6-diamidino-2-phenylindole (DAPI). Cells were sorted and analyzed using the Influx cell sorter (BD) and LSRII benchtop analyzer (BD).

Spheres and rudiments were stained for Keratin 8 (DHSB cat. no. TROMA-I, 1:100), Keratin 14 (BioLegend cat. no. 905301, 1:1000), GFP (abcam cat. no. ab5450, 1:500), and DAPI and analyzed on an LSM710 scanning confocal microscope (Zeiss). Formalin fixed, paraffin-embedded sections were stained with K8, K14, and RFP (MB Intl., PM005, 1:400) following citrate buffer-mediated antigen retrieval.

### In vitro sphere-forming assays

Cells were plated in 2% Matrigel-containing maintenance media (DMEM F12 with horse serum, hydrocortisone C, Cholera toxin, Insulin, ciprofloxacin, B27 supplement, and EGF), or differentiation media (Epicult B media containing B supplement [STEMCELL Technologies], Heparin, penicillin/streptomycin, EGF, and FGF) in low-adhesion 96-well plates. Spheres with diameters measuring 20 μM or greater were counted 7 days later. The percent of SFCs was determined as the number of spheres formed per cell plated. At least three wells were plated for each experiment to obtain technical repeats that were averaged. Each experiment was performed at least three times to obtain biological replicates. Two-tailed Student’s *t-*tests on averages of biological replicates were performed to determine *p-*values. *p-*values smaller than .05 were determined to be significant. For FACS analysis and serial passaging, colonies were isolated by incubating in cell recovery solution (Corning) on ice for 1.5 h and dissociated with Dispase, DNase, and .25% Trypsin-EDTA before being passed through a .40-μM filter and resuspended in Hank’s balanced salt solution containing 2% FBS and DNase. Diptheria toxin (DTx, Sigma) added to maintenance media with 2% Matrigel prior to plating fMaSCs.

### In vivo mammary repopulating assays

Endogenous epithelium was removed from the number 4 glands of 21-day-old CB-SCID mice and freshly isolated mammary cell suspensions were injected in limiting dilution with 1:1 matrigel and 1 μL .2% Trypan/PBS. Glands were isolated 8 weeks later and carmine stained to identify positive outgrowths. ELDA was carried out to statistically determine the number of MRU for a given cell population.^30^ Subsets of glands were then serially transplanted through single-cell suspension as stated above.

### Lineage tracing

For embryonic labeling, pregnant females were injected with .5 mg 4OHT (Sigma) dissolved in corn oil (Sigma) at gestation day E17. Resulting pups were aged to 8 weeks and glands were analyzed for tdTomato expression. For adult labeling, virgin female mice were injected once with 1.5 mg 4OHT and analyzed 8 weeks later. Population percentages are reported as the mean of at least four mice. Methods for isolation and staining for mammary epithelium for lineage-tracing experiments are the same as stated above.

### RT-PCR

mRNA transcripts were measured with Taqman assays using probes against murine Diptheria Toxin Receptor (DTR) (Mm00439306), Hprt (Mm01545399), Lgr4 (Mm00554385_m1), and Lgr5 (Mm00438890_m1) (Applied Biosciences). Means of Ct values over three experimental replicates are reported.

## Electronic supplementary material

Supplemental Figure Legends

Supplemental Material

Supplementary Figure S1

Supplementary Figure S2

Supplementary Figure S3

Supplementary Figure S5

Supplementary Figure S4

Supplementary Figure S6
