# Supplementary material for: Lgr5 is a marker for fetal mammary stem cells, but is not essential for stem cell activity or tumorigenesis
Source: NPJ Breast Cancer. 2017 Apr 24;3:16. doi: 10.1038/s41523-017-0018-6 (PMC5460261; doi:10.1038/s41523-017-0018-6)
Supplement: Supplementary file 1 — Supplemental Figure Legends [file 41523_2017_18_MOESM1_ESM.docx]

**Supplemental Figures**

**Supplemental Figure 1. Spheres from Lgr5-expressing fMaSCs have distinct phenotypes depending on growth media.**

EpCAM^HIGH^; CD49f^HIGH^; GFP^POS^ fMaSCs from E17 Lgr5KI embryos were plated in either serum based maintenance media or differentiation media supplemented with 2% Matrigel. Resulting spheres were isolated 7 days later, fixed, and stained for Keratin 14 (K14, red), Keratin 8 (K8, green), and DAPI (blue). Bar represents 100 μM. White arrow indicates a cell co-staining with K14 and K8. Images are representatives of 20 spheres over three separate experiments.

**Supplemental Figure 2. PCR verification of heterozygosity in Lgr5KI and Lgr4KI mice.**

A. Targeted genotyping of the Lgr5KI allele and exon 1 of the wild type allele was conducted on Lgr5KI^HET^ and Lgr5KI^HOM^ embryos. Lgr5KI^HET^ samples have both the Lgr5KI and wild type alleles (174 and 298 base pairs, respectively), and Lgr5KI^HOM^ embryos lack a wild type allele.

B. Targeted genotyping of the Lgr4 knock in (KI) and wildtype allele was conducted on Lgr4KI^HET^ and Lgr4KI^HOM^ embryos. Lgr4KI^HET^ samples have both the Lgr4KI and wild type alleles (384 and 185 base pairs, respectively), and Lgr4KI^HOM^ embryos lack a wild type allele.

**Supplemental Figure 3.**

**The Lgr5 promoter is not active in tumors resulting from C3(1)Tag; Lgr5KI^HOM^ and C3(1)Tag; Lgr5KI^HET^ fMaSCs.**

Tumors were harvested from mice, sectioned, and immunostained for GFP (green) and DAPI (blue). Successful staining was verified with intestine from Lgr5KI^HET^ adult mice, which is abundant in Lgr5-expressing cells.

**Supplemental Figure 4.**

**Lgr5 expression is acquired in out-growths resulting from GFP^NEG^ fMaSCs.**

Single cell suspensions were prepared from Lgr5KI^HET^ mice at stage E18. 4000 live, single, EpCAM^HIGH^; CD49f^HIGH^; GFP^POS^ and GFP^NEG^ cells were sorted and injected into cleared fat pads of recipient mice. 8 weeks later, glands were isolated, processed into single cell suspensions, and analyzed by FACS for expression of GFP, EpCAM, and CD49f. Suspensions generated from endogenous glands from wild type and Lgr5KI mice were used to set GFP gates and as a positive control, respectively.

**Supplemental Figure 5.**

**Diptheria toxin inhibits sphere formation in fMaSCs due to endogenous expression of the Diptheria toxin receptor.**

A. EpCAM^HIGH^; CD49f^HIGH^ fMaSCs were isolated from wild type CD1 mice and plated in maintenance media containing a dilution series of Diptheria Toxin (DTx). After 7 days, spheres were quantified. Percent SFC is reported as averages and error bars represent standard deviations from 3 technical replicates per dose.

B. RT-PCR was performed to detect endogenous expression of the Diphtheria Toxin Receptor (DTr) in wild type fMaSCs. Fold change relative to the Hprt housekeeping gene was calculated using Ct values and is reported as averages of three technical replicates.

**Supplemental Figure 6.**

**Lgr4 is not required for mammary rudiment development.**

A. RT-PCR was performed on fMaSCs isolated from Lgr5KI^HET^ and Lgr5KI^HOM^ embryos to detect expression of Lgr4 and Lgr5. Expression is reported as Ct values of the genes of interest minus the Ct values of the Gapdh housekeeping gene. Reported values represent averages of three technical replicates.

B. Fetal mammary rudiments were isolated from Lgr4KI^HOM^ embryos at stage E18 and stained in whole mount for Keratin 8 (K8, green) and Keratin 14 (K14, red).

C. Single cell suspensions were prepared from Lgr4KI^HET^ and Lgr4KI^HOM^ littermates at stage E18. Live, single, epithelial cells were analyzed for EpCAM and CD49f expression by FACS.

D. Single cell suspensions were prepared from Lgr4KI^HET^ and Lgr4KI^HOM^ littermates at stage E18. Live, single, EpCAM^HIGH^; CD49f^HIGH^; cells were sorted and plated in to 3D culture containing 2% Matrigel. After one week, spheres were quantified and percent sphere forming cells was determined by the number of spheres counted per the number of cells plated.
