# Supplementary material for: Lgr5 is a marker for fetal mammary stem cells, but is not essential for stem cell activity or tumorigenesis
Source: NPJ Breast Cancer. 2017 Apr 24;3:16. doi: 10.1038/s41523-017-0018-6 (PMC5460261; doi:10.1038/s41523-017-0018-6)
Supplement: Supplementary file 2 — Supplemental Material [file 41523_2017_18_MOESM2_ESM.docx]

**Lgr4 is not required for fetal mammary development or stem cell activity**

Lgr5 is part of a family that also includes Lgr4 and 6, both known to play roles in stem cell biology^35^. Importantly, Lgr4 has been shown to have a regulatory role in mammary stem cell activity^6, 32^. We used RT-PCR to determine if Lgr4 may compensate for loss of Lgr5. First, we verified that Lgr4 was expressed in EpCAM^HIGH^; CD49f^HIGH^; GFP^POS^ fMaSCs isolated from E18 Lgr5KI^Het^ and Lgr5^HOM^ embryos, but saw no difference in expression of Lgr4 between the genotypes (*p* = .33). As expected, Lgr5 mRNA was not detected in Lgr5^HOM^ fMaSCs (Fig. S6 A).

We next tested the functional requirement of Lgr4 in the embryonic gland using an Lgr4KI model in which mCherry is inserted immediately downstream of the Lgr4 promoter, effectively inactivating the endogenous gene^33^. We generated Lgr4KI^HOM^ embryos and verified the genotypes of individual embryos with PCR (Fig. S2B). Rudiments isolated from Lgr4KI^HOM^ embryos had no difference in morphology compared to heterozygous and wild type littermates. We also noted abundant cells staining positive for both K8 and K14 (Fig S6 B). We prepared single cell suspensions from Lgr4KI^HET^and Lgr4^HOM^ littermates and saw a comparable EpCAM^HIGH^; CD49f^HIGH^ population (Fig. S6 C). EpCAM^HIGH^; CD49f^HIGH^ cells from Lgr4KI^HET^ and Lgr4^HOM^ littermates formed spheres at a frequency that was not significantly different (14% vs. 13%, *p* = .66), (Fig. S6 D). These experiments clearly show that similar to Lgr5, Lgr4 by itself is not required for fetal rudiment development or stem cell activity.
