# Supplementary figures and images for: Lgr5 is a marker for fetal mammary stem cells, but is not essential for stem cell activity or tumorigenesis
Source: NPJ Breast Cancer. 2017 Apr 24;3:16. doi: 10.1038/s41523-017-0018-6 (PMC5460261; doi:10.1038/s41523-017-0018-6)

Figure S1.

Maintenance media

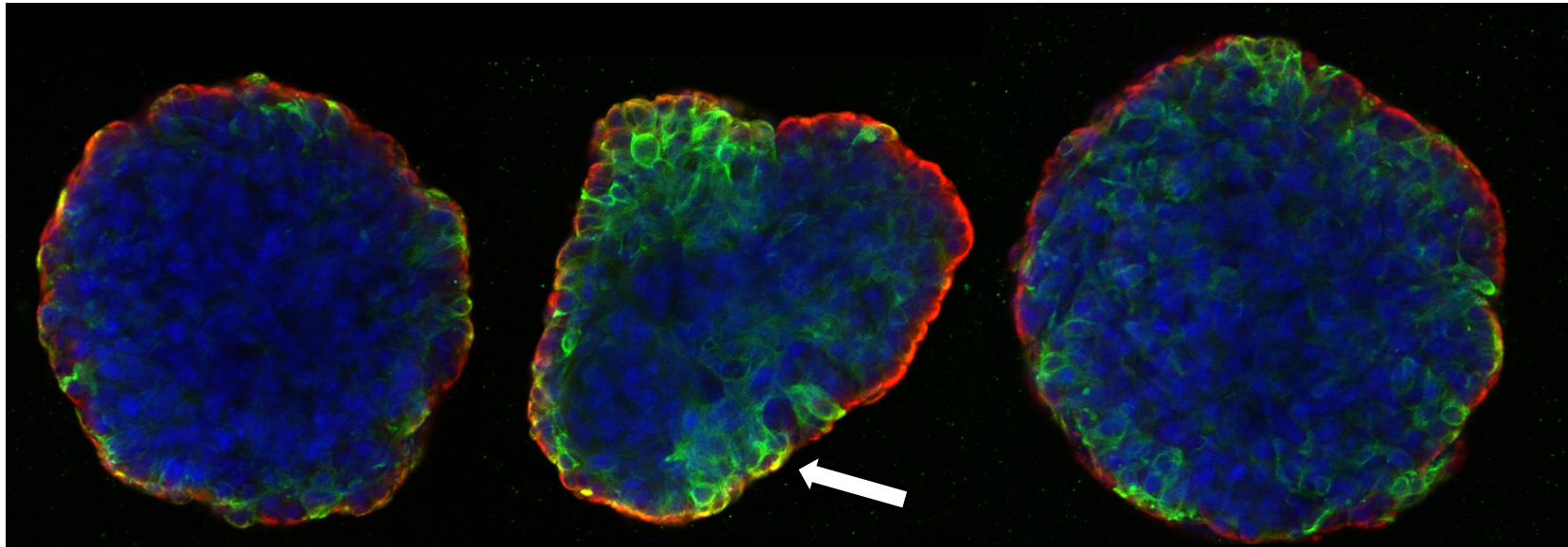

Differentiation media

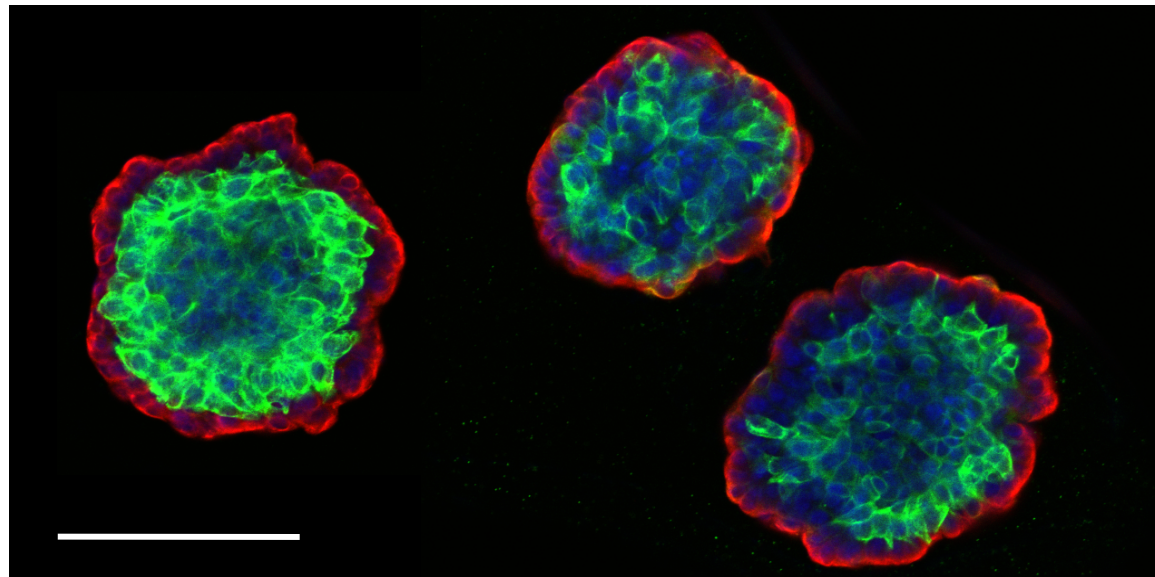

K8  
K14  
DAPI

Supplement: Supplementary file 3 — Supplementary Figure S1 [file 41523_2017_18_MOESM3_ESM.pdf]

Figure S2.

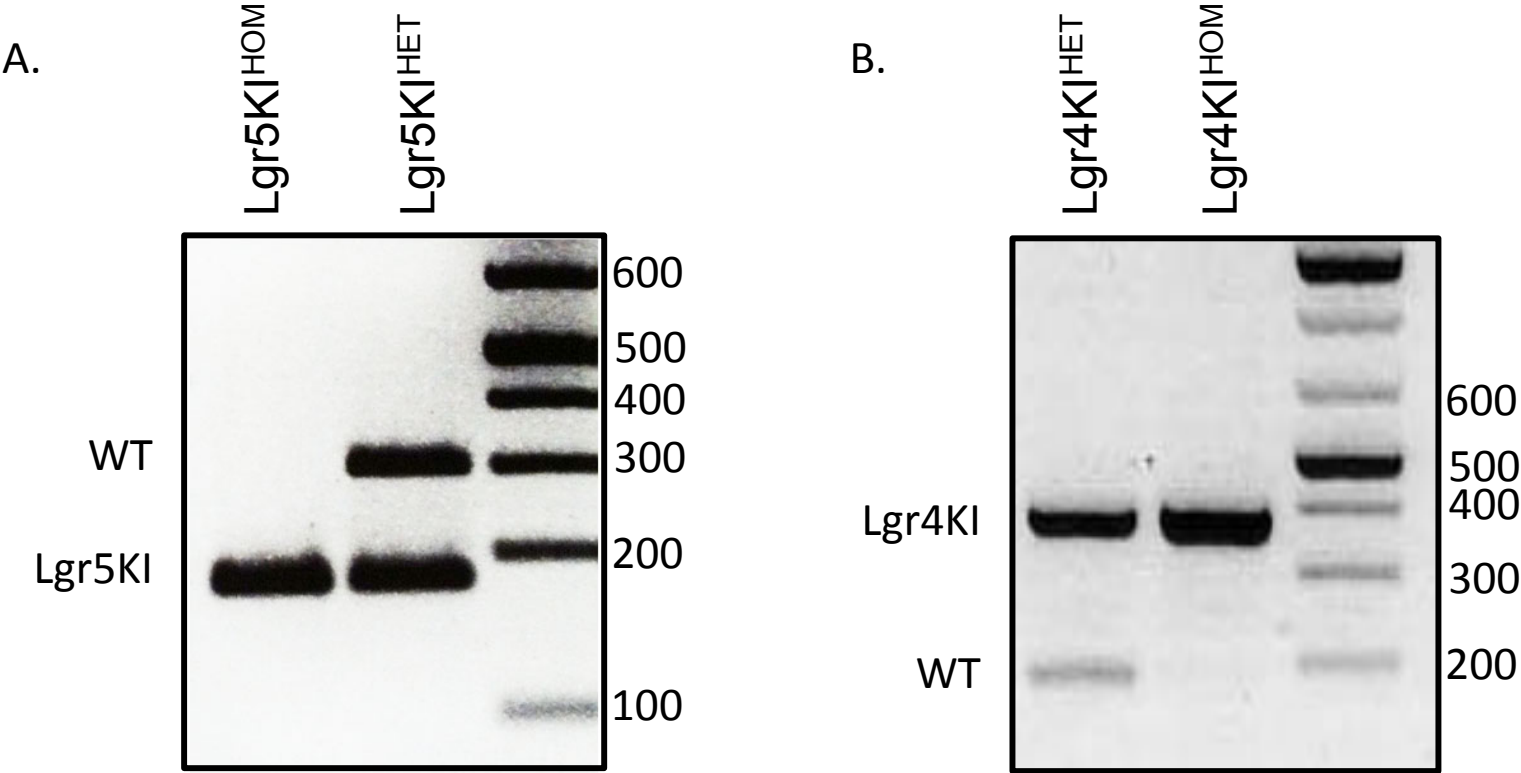

Supplement: Supplementary file 4 — Supplementary Figure S2 [file 41523_2017_18_MOESM4_ESM.pdf]

Fig. S3

GFP  
DAPI

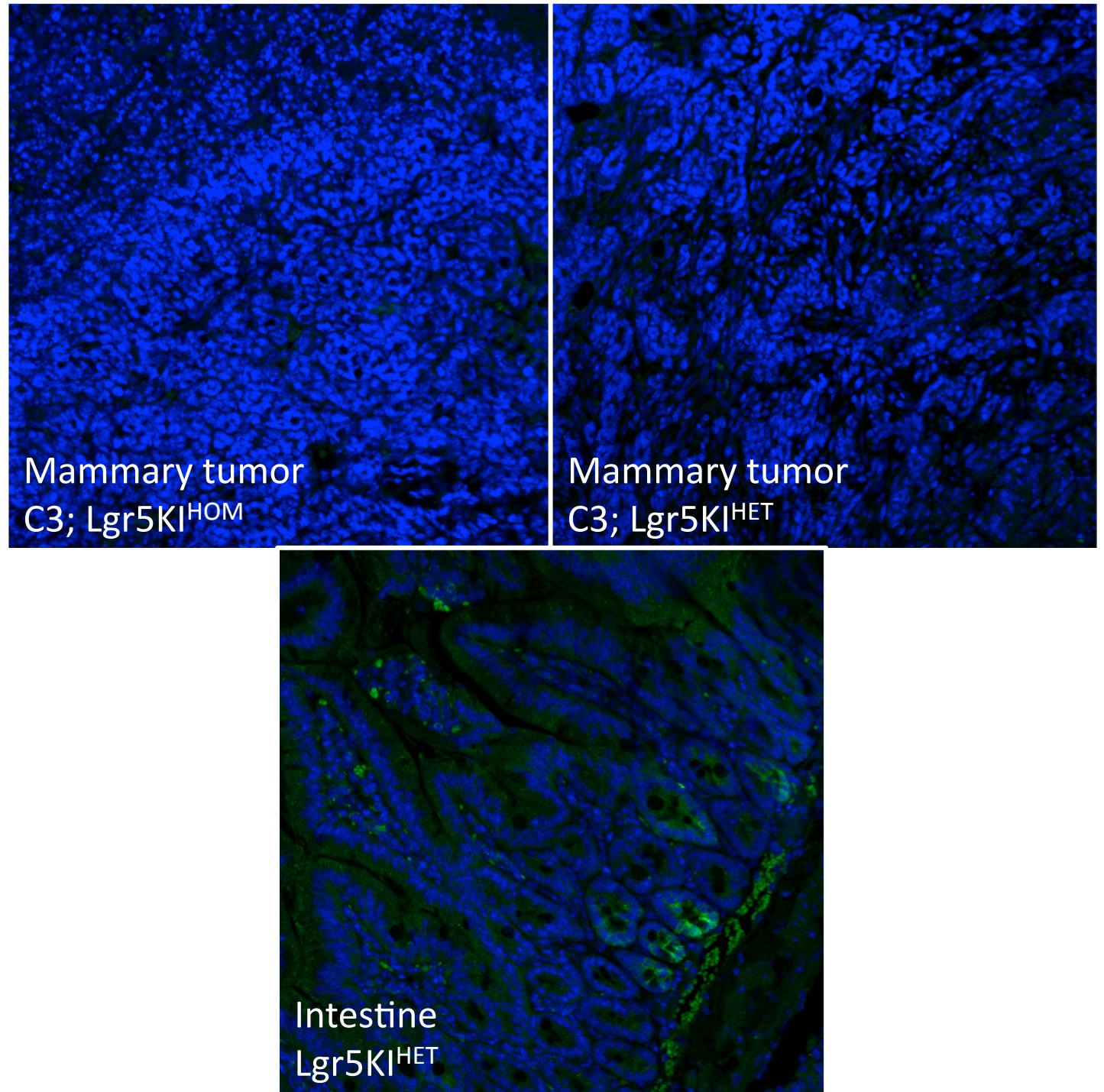

Supplement: Supplementary file 5 — Supplementary Figure S3 [file 41523_2017_18_MOESM5_ESM.pdf]

Figure S5.

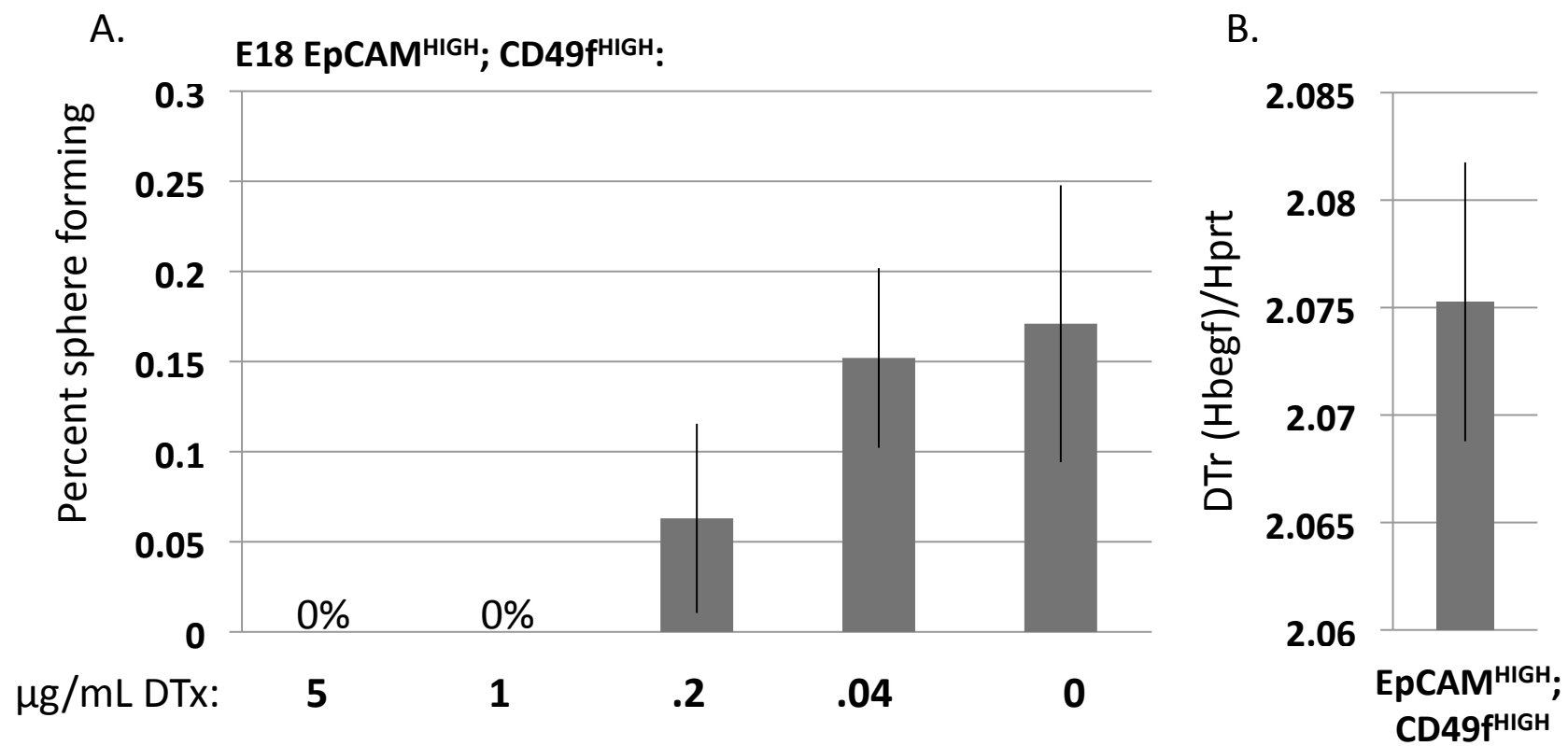

Supplement: Supplementary file 6 — Supplementary Figure S5 [file 41523_2017_18_MOESM6_ESM.pdf]

Figure S6.

A.

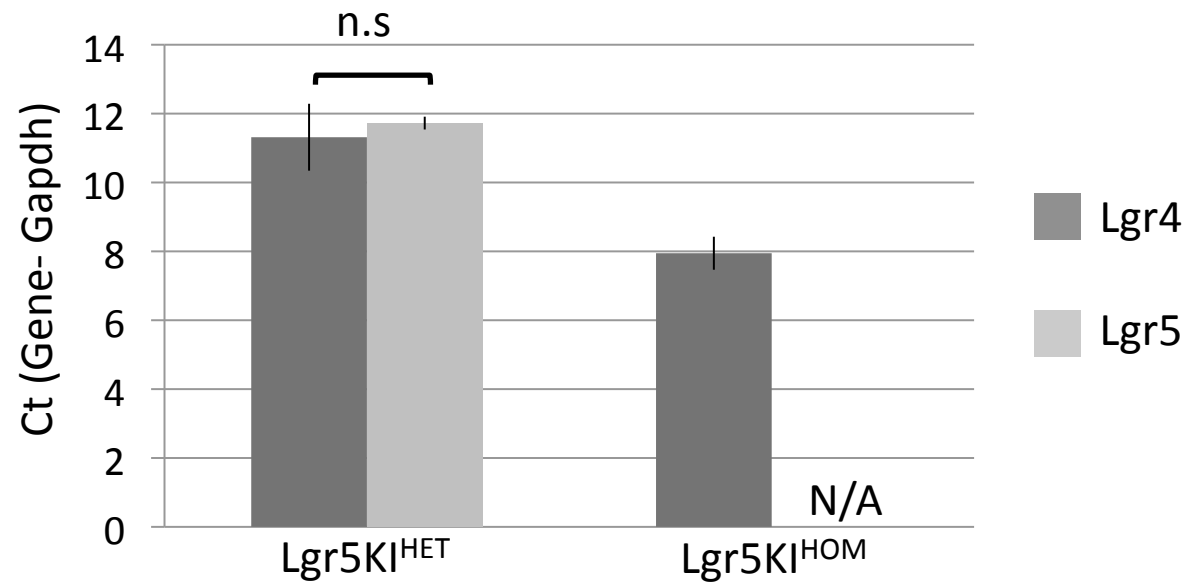

B.

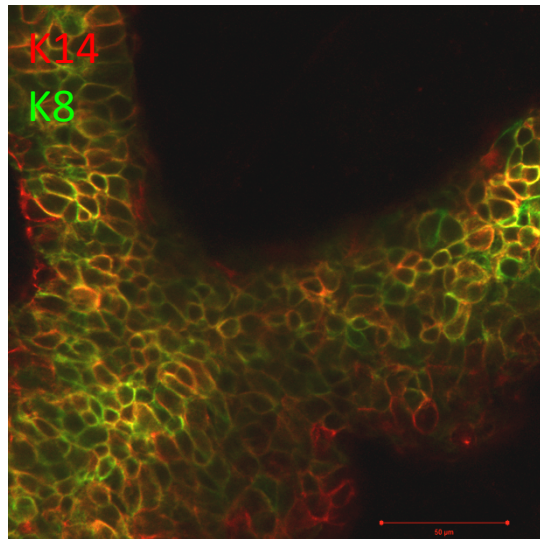

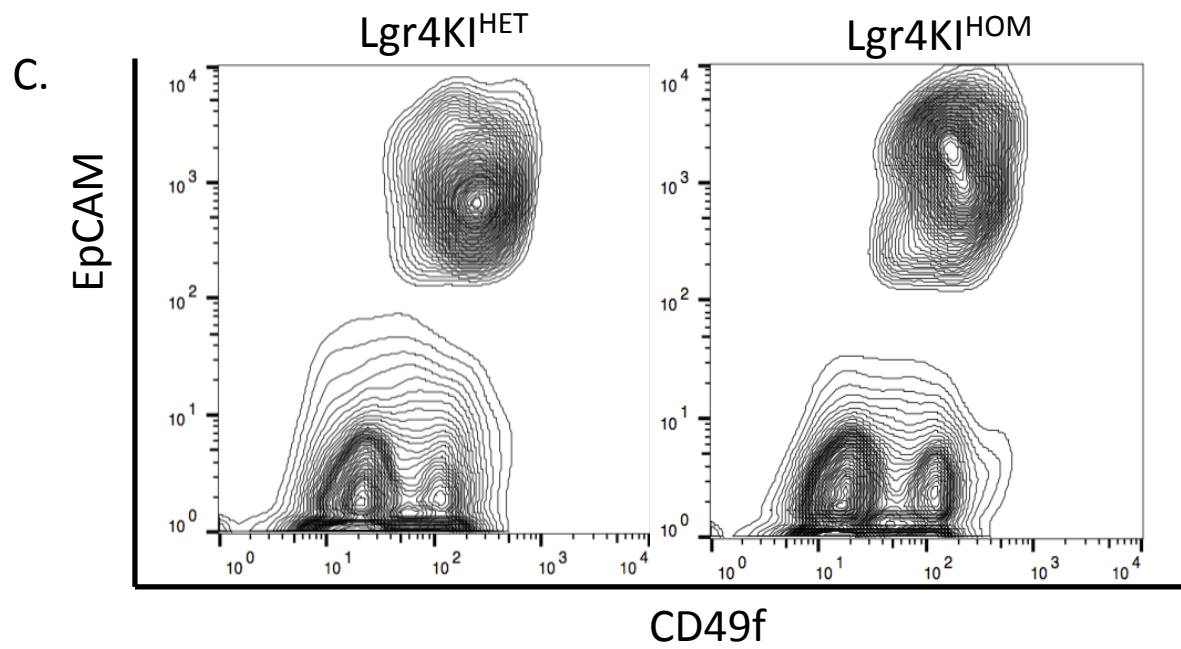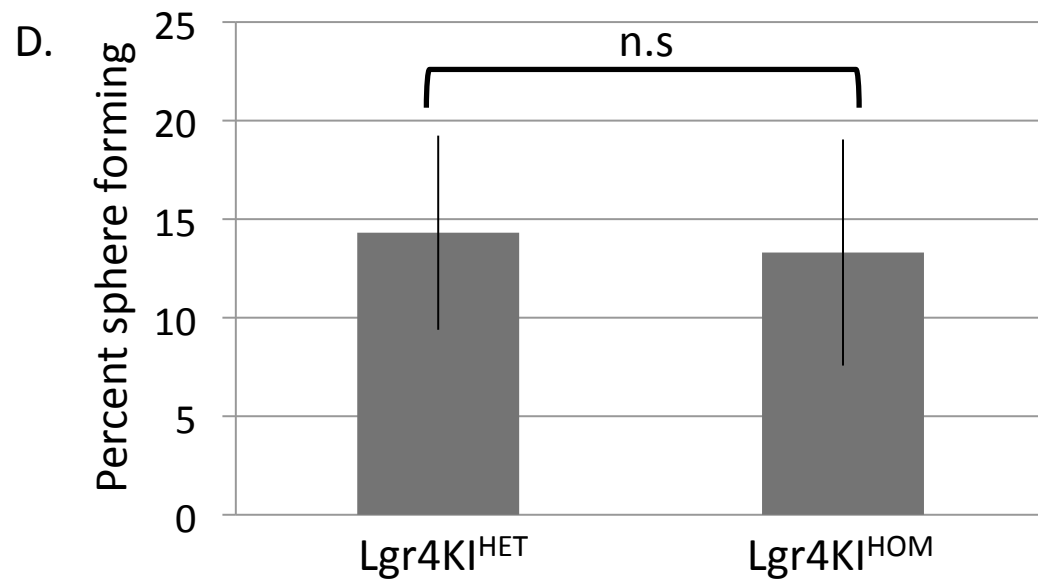

Supplement: Supplementary file 8 — Supplementary Figure S6 [file 41523_2017_18_MOESM8_ESM.pdf]
