# Supplementary material for: Lgr5 is a marker for fetal mammary stem cells, but is not essential for stem cell activity or tumorigenesis
Source: NPJ Breast Cancer. 2017 Apr 24;3:16. doi: 10.1038/s41523-017-0018-6 (PMC5460261; doi:10.1038/s41523-017-0018-6)
Supplement: Supplementary file 7 — Supplementary Figure S4 [file 41523_2017_18_MOESM7_ESM.pdf]

Figure S4.

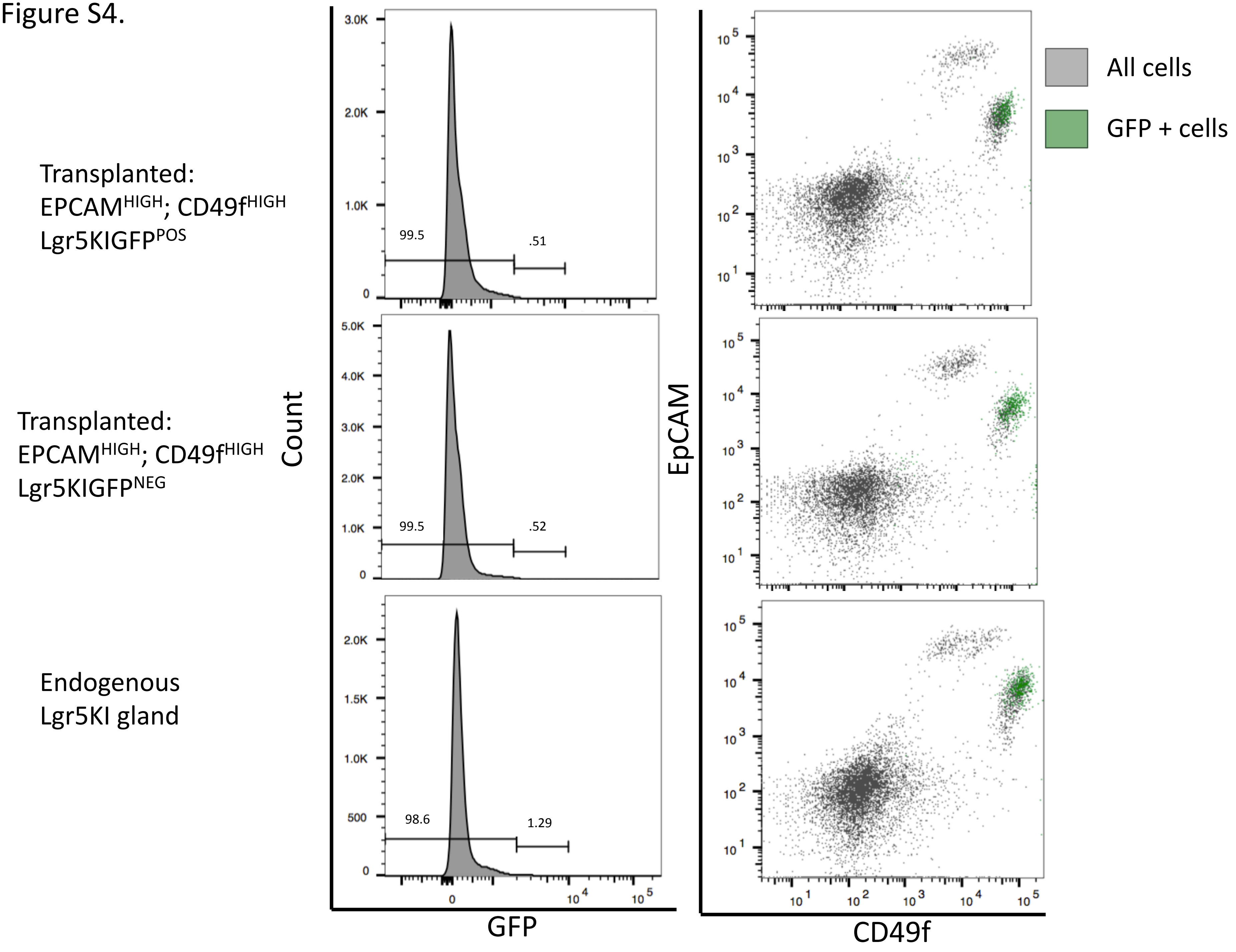
Transplanted:
EPCAMHIGH; CD49fHIGH
Lgr5KIGFPPOS
Transplanted:
EPCAMHIGH; CD49fHIGH
Lgr5KIGFPNEG
Endogenous
Lgr5KI gland
Count
99.5
.51
99.5
.52
98.6
1.29
GFP
EpCAM
CD49f
All cells
GFP + cells
